# Supplementary figures and images for: Molecular Characterization of a Recombinant Isolate of Tomato Leaf Curl New Delhi Virus Associated with Severe Outbreaks in Zucchini Squash in Southern Italy
Source: Plants (Basel). 2023 Jun 21;12(13):2399. doi: 10.3390/plants12132399 (PMC10346446; doi:10.3390/plants12132399)

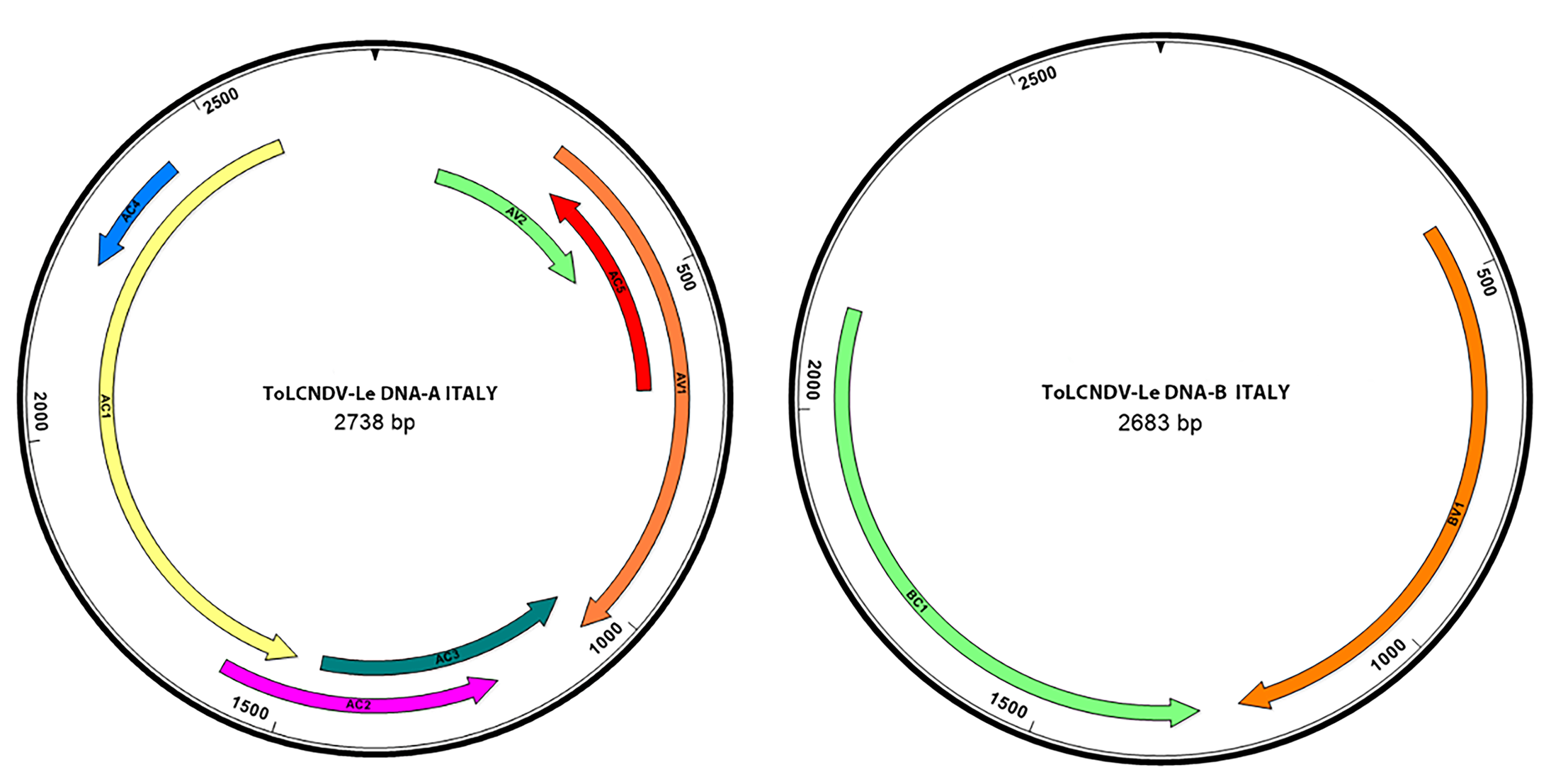

Supplement: Supplementary file 1 [file plants-12-02399-s001.zip › Figure S1.tif]

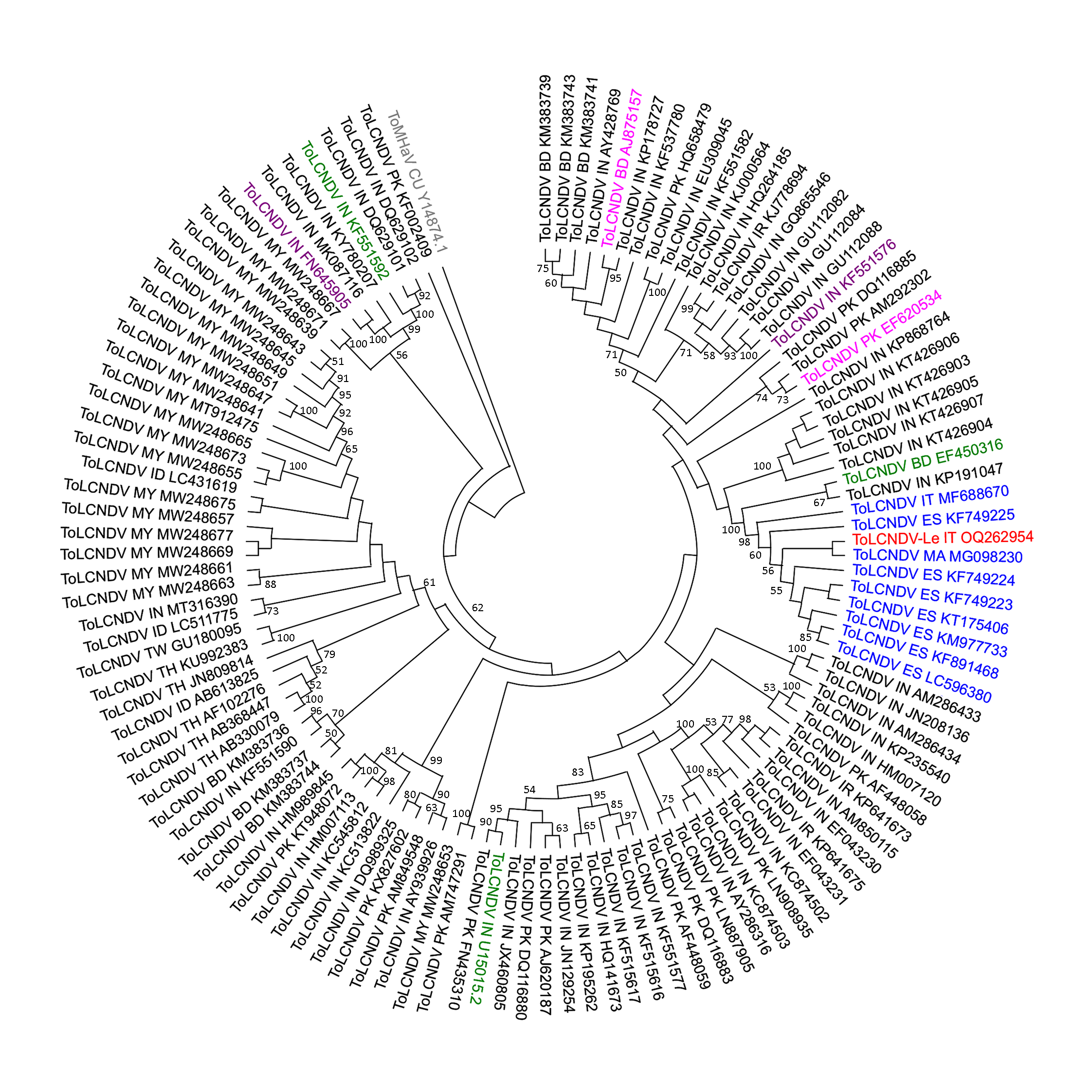

Supplement: Supplementary file 1 [file plants-12-02399-s001.zip › Figure S2.tif]

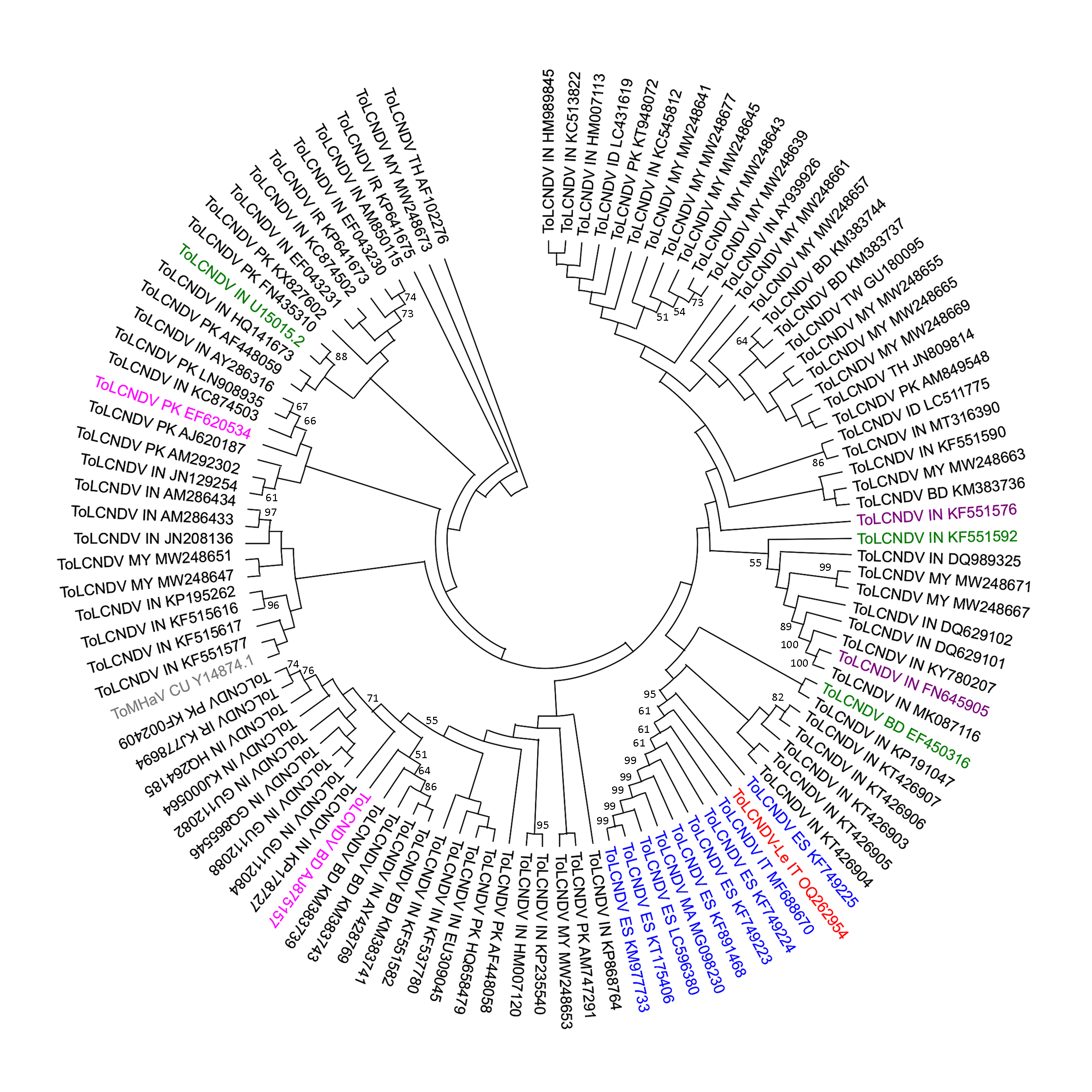

Supplement: Supplementary file 1 [file plants-12-02399-s001.zip › Figure S3.tif]
